# Karyotypic similarities between two species of *Rhamphichthys* (Rhamphichthyidae, Gymnotiformes) from the Amazon basin

**DOI:** 10.3897/CompCytogen.v7i4.4366

**Published:** 2013-10-24

**Authors:** Patrícia Corrêa da Silva, Cleusa Yoshiko Nagamachi, Danillo dos Santos Silva, Susana Suely Rodrigues Milhomem, Adauto Lima Cardoso, Jonas Alves de Oliveira, Julio Cesar Pieczarka

**Affiliations:** 1 Universidade Federal do Pará, Instituto de Ciências Biológicas, Av Perimetral, SN. Guamá. 66075-900. Belém, Para, Brazil; 2 CNPq Researcher, Belém, Brazi; 3 CNPq Mastership Scholarship in Genetics and Molecular Biology; 4 CNPq Doctorship Scholarship in Genetics and Molecular Biology; 5 CNPq Undergraduated Scholarship; 6 Instituto de Desenvolvimento Sustentável Mamirauá, Estrada do Bexiga, 2584 69470-000. Tefé, Amazonas, Brazil

**Keywords:** Gymnotiformes, Rhamphichthyidae, Cytogenetics, FISH

## Abstract

The family Rhamphichthyidae includes three genera: *Rhamphichthys* Müller et Troschel, 1846, *Gymnorhamphichthys* M. M. Ellis, 1912 and *Iracema* Triques, 1996. From this family, only the species *Rhamphichthys hanni* Meinken, 1937 has had its karyotype described. Here, we describe the karyotypes of two additional *Rhamphichthys* species: *Rhamphichthys marmoratus* Castelnau, 1855 from the Reserva de Desenvolvimento Sustentável Mamirauá, Amazonas state and *Rhamphichthys* prope *rostratus* Linnaeus, 1766 from Pará state, both in Brazil. Our karyotypic analyses demonstrated that the diploid number is conserved for the genus (2n = 50), but the karyotypic formulas (KFs) differed between *Rhamphichthys marmoratus* (44m/sm+6a) and *Rhamphichthys* prope *rostratus* (42m/sm+8a). In both species, the constitutive heterochromatin (CH) was located in the centromeric region of most chromosomes. Large heterochromatic blocks were found on the long arms of pairs 4 and 14 in *Rhamphichthys marmoratus* and on chromosomes 3, 4 and 19 in *Rhamphichthys* prope *rostratus*, which also has a heteromorphism in chromosome pair 1. The CH was DAPI positive, indicating that it is rich in AT base pairs. The Nucleolus Organizer Region (NOR) showed staining at a single location in both species: the long arm of pair 1 in *Rhamphichthys marmoratus* and the long arm of pair 12 in *Rhamphichthys* prope *rostratus*, where it showed a size heteromorphism. CMA_3_ staining coincided with that of Ag-NOR, indicating that the ribosomal genes contain interspaced GC-rich sequences. FISH with an 18S rDNA probe confirmed that there is only one NOR site in each species. These results can be used as potential cytogenetic markers for fish populations, and comparative analysis of the karyotypes of *Hypopygus* Hoedman, 1962, *Rhamphichthys* and *Steatogenys* Boulenger, 1898 suggests that the first two genera diverged later that the third.

## Introduction

The family Rhamphichthyidae comprises three genera: *Rhamphichthys* Müller et Troschel, 1846, with eight described species, *Gymnorhamphichthys* Ellis, 1912, with six species, and *Iracema* Triques, 1996, with only one species (Ferraris 2003, Lundberg 2005, Triques 2005, Carvalho et al. 2011) (Table 1). These numbers are likely to be an underestimate, since the number of species described in Gymnotiformes has increased over the last 15 years (Albert and Crampton 2005).

**Table 1.** Species of Rhamphichthyidae (According to Ferraris 2003 and Albert and Crampton 2005).

**Species**	**Locality**
*Gymnorhamphichthys hypostomus* Ellis, 1912	São Joaquim, Bolivia
*Gymnorhamphichthys rondoni* Miranda Ribeiro, 1920	17 de Fevereiro River, Amazonas, Brazil
*Gymnorhamphichthys petiti* Géry et Vu-Tân-Tuê, 1964	Bananal Island, Araguaia River, Brazil
*Gymnorhamphichthys rosamariae* Schwassmann, 1989	Negro River, Amazonas, Brazil
*Gymnorhamphichthys bogardusi* Lundberg, 2005	Orinoco River, Delta Amacuro State
*Gymnorhamphichthys britskii* Carvalho et al., 2011	Paraná- Paraguay System
*Iracema caiana* Triques, 1996	Jauaperi Beach, Negro River, Amazonas, Brazil
*Rhamphichthys apurensis* Fernández-Yépez, 1968	Bucaral River, a tributary of Apure River, Venezuela
*Rhamphichthys atlanticus* Triques, 1999	Viana Lake, Amazonas, Brazil
*Rhamphichthys drepanium* Triques, 1999	Janauari Lake, confluence of the Negro and Solimões Rivers, Amazonas, Brazil
*Rhamphichthys hahni* Meinken, 1937	Paraná River basin, next to Corrientes, Argentina
*Rhamphichthys lineatus* Castelnau, 1855	Ucayali River basin, Peru
*Rhamphichthys longior* Triques, 1999	Paru Lake, confluence of the Trombetas River, Para, Brazil
*Rhamphichthys marmoratus* Castelnau, 1855	Araguaia River, Brazil; Ucayali River, Peru
*Rhamphichthys rostratus* Linnaeus, 1766	South America

The species of *Rhamphichthys* have a long and narrow body, a long tubular snout, no teeth in the jaw, and an anal fin with more than 300 rays. They are slow swimmers and spend most of their time at the bottoms of rivers (Mago-Leccia 1994, Ferraris 2003, Triques 2005). Among the Gymnotiformes, *Rhamphichthys* has the largest diversity and abundance in the Amazon basin, and the species *Rhamphichthys rostratus* Linnaeus, 1766 has the largest geographic distribution when compared with the other species of this genus (Ferraris 2003). All *Rhamphichthys* species generate electrical pulses that are used to communicate and identify mating partners and other species. This trait allows them to be nocturnal and live in rivers with dark waters (Kawasaki et al. 1996, Crampton 1998, Nanjappa et al. 2000, Gouvêa et al. 2002).

The phylogeny of the Gymnotiformes proposed by Albert (2001) was based on morphophysiological, behavioral and DNA sequence analyses by Alves-Gomes et al. (1995). In it, the families Rhamphichthyidae and Hypopomidae form a monophyletic group (Rhamphichthyoidea) that is separated from the clade that includes the families Sternopygidae and Apteronotidae. Among the Rhamphichthyoidea, the tribe Steatogenini (*Steatogenys* Boulenger, 1898, *Hypopygus* Hoedman, 1962 and *Stegostenopos* Triques, 1997) is accepted as monophyletic (Albert and Campos-da-Paz 1998, Crampton et al. 2007), but there is some debate as to whether this tribe belongs to the Rhamphichthyidae (Alves-Gomes et al. 1995) or the Hypopomidae (Albert 2001).

Relatively few cytogenetic studies have been performed in Gymnotiformes. According to Oliveira et al.(2009), only 48 species of this order have had their karyotypes described. The genera *Gymnotus* Linnaeus, 1758 and *Eigenmannia* Jordan et Evermann, 1896 have the most available information on their karyotypic diversity (Almeida-Toledo et al. 2001, 2002, Lacerda and Maistro 2007, Milhomem et al. 2007, 2008, Silva et al. 2009, Nagamachi et al. 2010).

In Rhamphichthyoidea, the available chromosome information comes from only six species (Table 2): *Hypopomus artedi* Kaup, 1856 with diploid number (2n) = 38, Fundamental Number (FN) = 70 and Karyotypic Formula (KF) = 32m/sm+6st/a; *Hypopygus lepturus* Hoedman, 1962with 2n = 50, FN = 86 and KF = 36m/sm+10st+4a; *Brachyhypopomus brevirostris* Steindachner, 1868, with 2n = 36, FN = 42 and KF = 6m/sm+30st/a (Almeida-Toledo et al. 2000); *Brachyhypopomus pinnicaudatus* Hopkins, 1991, with 2n = 41 in males and 42 in females (X_1_X_2_Y sex system) and FN = 42, with all acrocentric chromosomes except the Y (Almeida-Toledo 1978); *Steatogenys elegans* Steindachner, 1880, with 2n = 50 (ZZ/ZW sex system), FN = 62 and KF = 12m/sm+38st/a; *Steatogenys duidae* La Monte, 1929, with 2n = 50, FN = 100 and KF=50m/sm (Cardoso et al. 2011); and *Rhamphichthys hahni* Meinken, 1937, with 2n = 50, FN = 94 and FK = 44m/sm+6st/a (Mendes et al. 2012).

**Table 2.** A review of the cytogenetic information in Rhamphichthyoidea from Cardoso et al.(2011) with modifications.

**Family / Species**	**2n**	**KF**	**Sex system**	**CB**	**NOR**	**References**
**Hipopomidae**						
*Hypopomus artedi* Kaup, 1856	38	32m-sm / 6st-a	Absent	**-**	**-**	Almeida–Toledo (1978) in Oliveira et al. (2009)
*Brachyhypopomus brevirostris* Steindachner, 1868	36	6m-sm / 30st-a	Absent	**-**	**-**	Almeida–Toledo (1978) in Oliveira et al. (2009)
*Brachyhypopomus pinnicaudatus* (Hopkins, 1991)	41♂ / 42♀	1m/41a♂ / 42a♀	X1X2Y	Centromeric region of most chromosomes	Multiple	Almeida–Toledo et al. (2000)
*Hypopygus lepturus* Hoedeman, 1962	50	36m-sm / 14st-a	Absent	**-**	**-**	Almeida–Toledo (1978) in Oliveira et al. (2009)
*Steatogenys elegans* (Steindachner, 1880)	50	12m-sm/ 38st-a	ZZ/ZW	Centromeric region of all chromosomes and interstitial (1q and 2 blocks in Wq)	Single	Cardoso et al. (2011)
*Steatogenys duidae* (La Monte, 1929)	50	50 m-sm	Absent	Centromeric and pericentromeric region of all chromosomes and interstitial (2q , 3q, 5q and 7q)	Single	Cardoso et al. (2011)
**Rhamphichthyidae**						
*Rhamphichthys hahni* (Meinken, 1937)	50	44m-sm / 6a	Absent	Centromeric region of most chromosomes and blocks of CH in three chromosomes (SM)	Single	Mendes et al. (2012)
*Rhamphichthys marmoratus* Castelnau, 1855	50	44m-sm / 6st-a	Absent	Centromeric region of most chromosomes and interstitial blocks (4q and 14p)	Single	Present work
*Rhamphichthys* prope *rostratus* (Linnaeus, 1766)	50	42m-sm / 8a	Absent	Centromeric region of most chromosomes and interstitial blocks (3q, 4q and 19p)	Single	Present work

In the present work, we studied the karyotypes of two species of *Rhamphichthys* from the Amazon region in an effort to better define the boundaries between the species, and compared our findings with those from the single previously described species of *Rhamphichthys* to better understand the phylogenetic relationships in this genus.

## Material and methods

**Figure 1.**
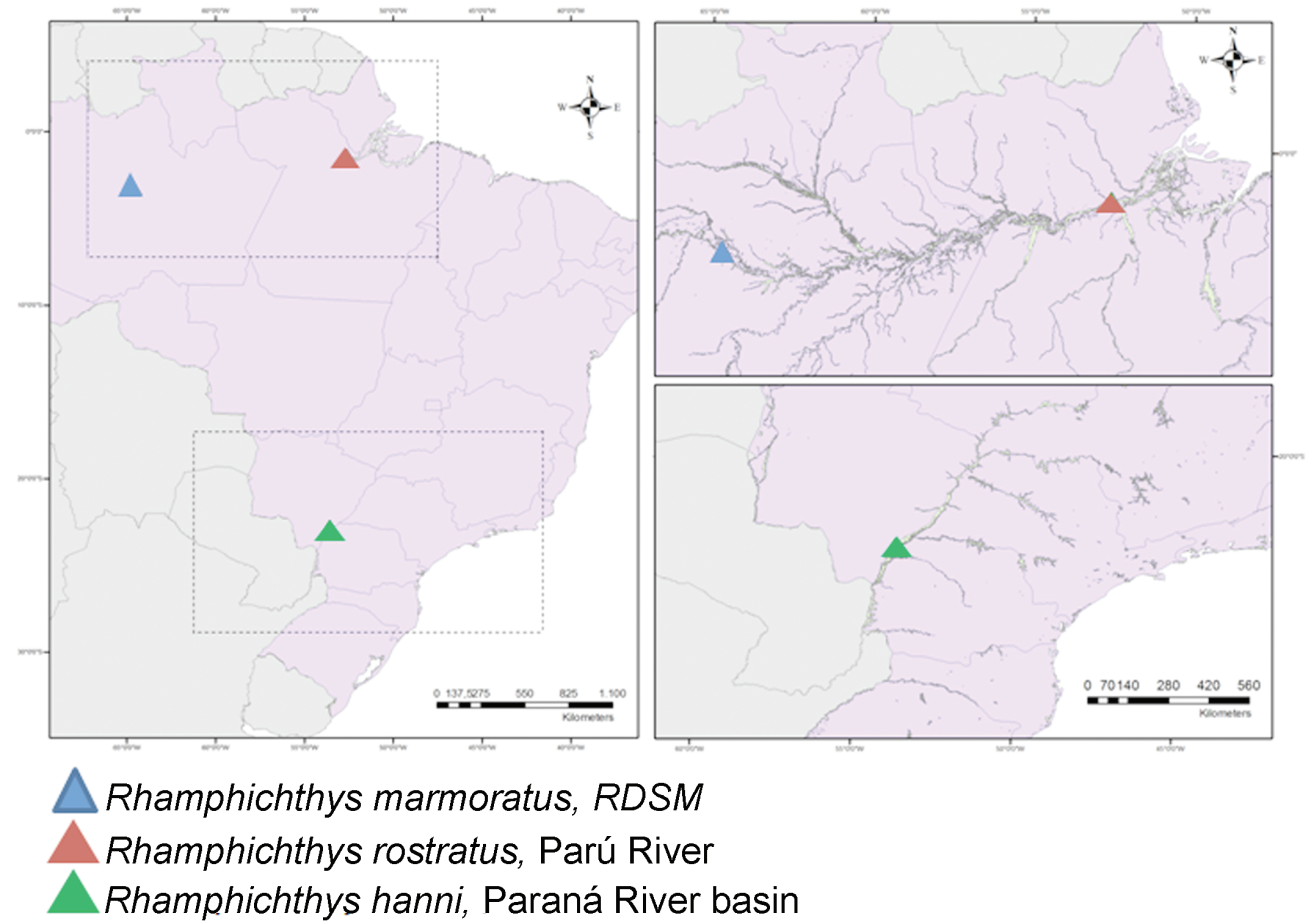
A map with the location of the *Rhamphichthys* species with cytogenetic descriptions. *Rhamphichthys marmoratus* and *Rhamphichthys rostratus* were analyzed in the present work.

Fishes were collected using a bioamplification device that detects electric fields and translate them into sounds (Crampton et al. 2007). We analyzed 13 animals (seven males and six females) of *Rhamphichthys marmoratus* Castelnau, 1855, collected from rivers in the Reserva de Desenvolvimento Sustentável Mamirauá (Mamirauá Sustainable Development Reserve, RSDM), Amazonas state, Brazil (03°07'32.5"S, 064°46'47.3"W). The sample was deposited in the museum of the RSDM (IDSMIctio000735 and IDSMIctio000750). The two individuals of *Rhamphichthys* prope *rostratus* Linnaeus, 1766, one male and one female, came from the Parú River, Pará state, Brazil (01°31'13.39"S, 52°38'49.00"W). This sample was deposited in the Museu Paraense Emílio Goeldi (MPEG 18347). Figure 1 shows the collection sites.

Metaphase chromosomes were obtained according to the method described by Bertollo et al. (1978) and analyzed by Giemsa staining, C-banding (Sumner 1972), Ag-NOR staining (Howell and Black 1980), CMA_3_ banding (Schweizer 1980) and DAPI banding (Pieczarka et al. 2006). Fluorescent *In Situ* Hybridization (FISH) was performed using 18S rDNA probes from *Prochilodus argenteus* Spix et Agassiz, 1829 (Hatanaka and Galetti Jr 2004). Microscopic images were obtained using a Zeiss Axiophot 2 microscope and a Zeiss Axiocam Mrm controlled by the Zeiss Axiovision software. Metaphase organization was performed following the method of Levan et al. (1964).

## Results

### Rhamphichthys marmoratus

**Figure 2.**
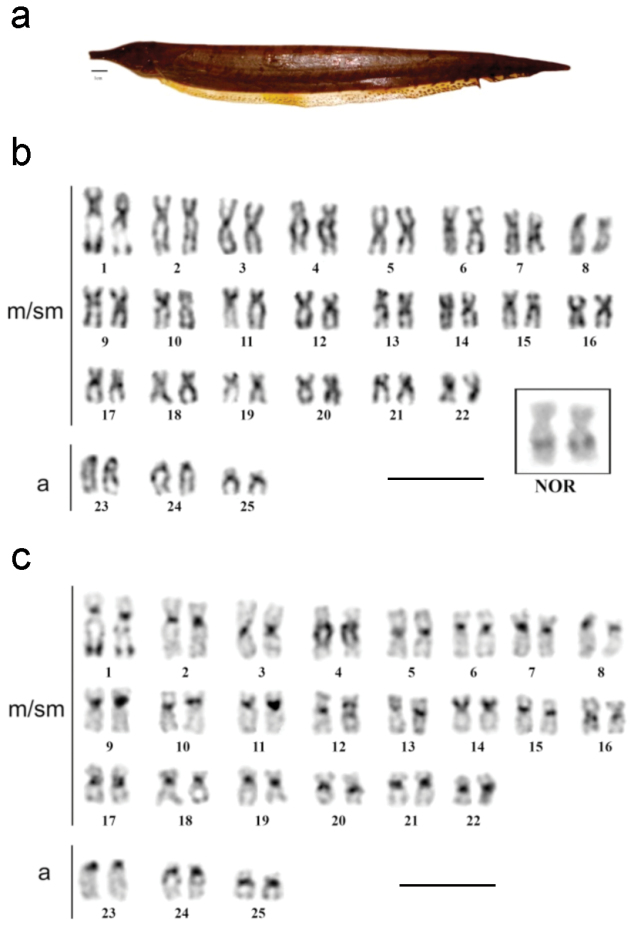
**a** *Rhamphichthys marmoratus* **b** Giemsa stained karyotype with the NOR bearer pair into the box **c** C-banded sequenced karyotype (m/ms- metacentric/submetacentric, a- acrocentric). Scale bar: **a**) 1 cm, **b**) and **c**) 10 μm.

**Figure 3.**
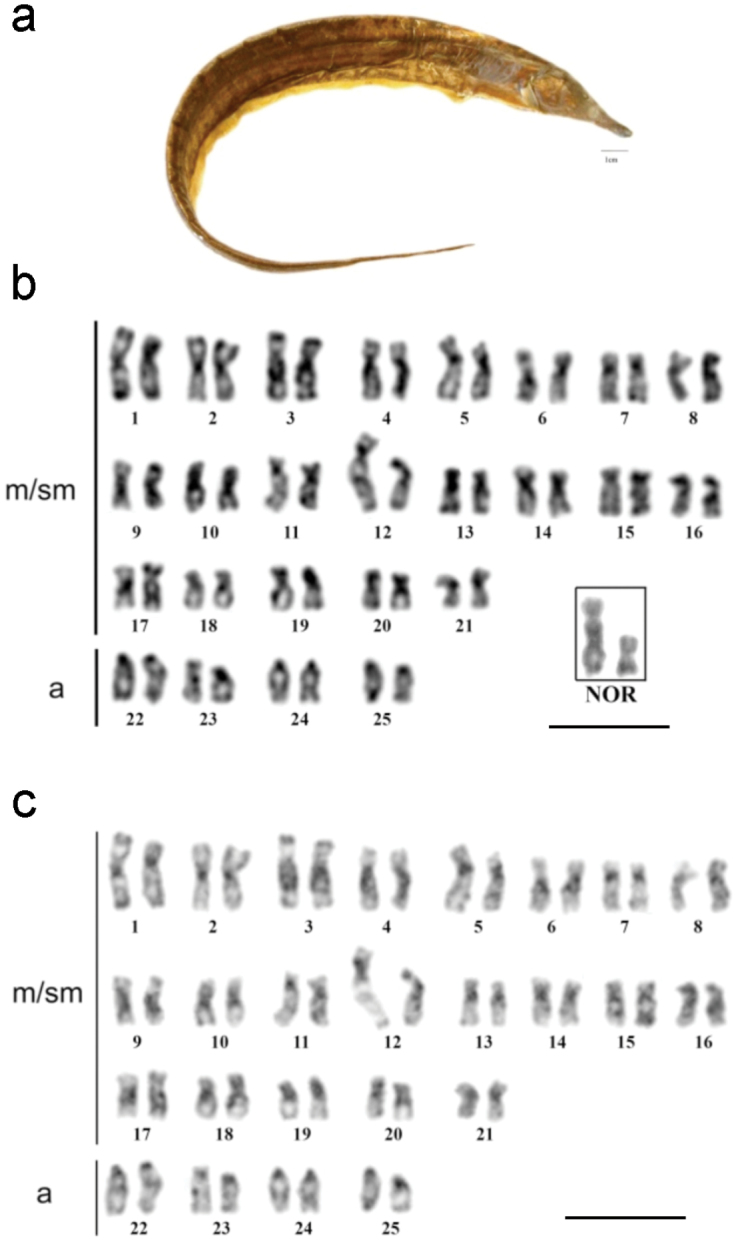
**a** *Rhamphichthys* prope *rostratus* **b** Giemsa stained karyotype with the NOR bearer pair into the box **c** C-banded sequenced karyotype; (m/ms- metacentric/submetacentric, a- acrocentric). Scale bar: **a**) 1 cm, **b**) and **c**) 10 μm.

All samples of *Rhamphichthys marmoratus* (Fig. 2) had 2n = 50 and a karyotypic formula (KF) consisting of 44 metacentric/submetacentric (m/sm) and 6 acrocentric chromosomes (Fig. 2a), with no evidence of any sex-determination chromosome system. Ag-NOR staining showed that the NOR is located in the interstitial region of the long arm of pair 1, in a secondary constriction (Fig. 2b, box). Constitutive heterochromatin (CH) was found in the centromeric regions of all chromosomes (Fig. 2c). Pair 4 was notable for a large heterochromatic block running from the proximal region across most of the long arm, while pair 14 had a CH block covering most of its short arm. CH was also found in the distal region of the long arm of pair 1 (Fig. 2c). DAPI fluorochrome banding coincided with positive C-banding in all centromeres, and was especially strong in pairs 4 (Fig. 3a). The CMA_3_ fluorochrome banding localized to the same region as the NOR, suggesting that this region is GC-rich (Fig. 3b). FISH with 18S rDNA probes confirmed that the NOR is located in the interstitial region of the long arm of pair 1 (Fig. 3c).

### *Rhamphichthys* prope *rostratus*

**Figure 4.**
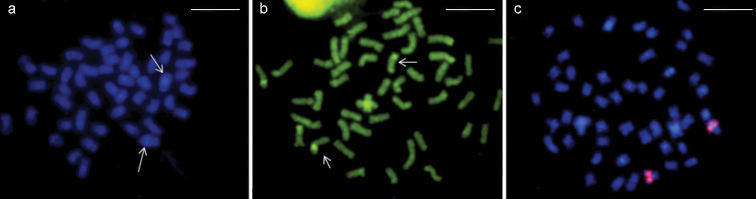
*Rhamphichthys marmoratus* - **a** DAPI staining. Arrows: pair 4 with a large CH block **b** CMA_3_ staining, arrows designate NOR pair **c** FISH with rDNA probe. Scale bar: 10 μm.

**Figure 5.**
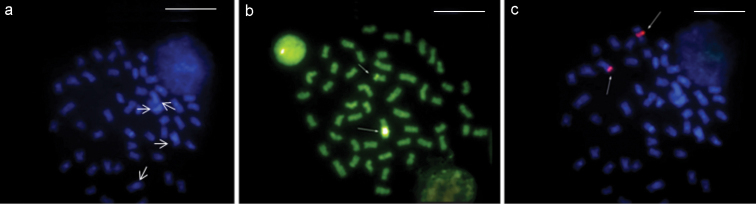
*Rhamphichthys rostratus* - **a** DAPI staining, arrows designate pairs 3 and 4 with large CH blocks **b** CMA_3_ staining, arrows designate NOR pair **c** FISH with rDNA probe. Scale bar: 10 μm.

*Rhamphichthys* prope *rostratus* (Fig. 4a) had 2n = 50 and a KF of 42m/sm+8a, with no evidence of a sex-determination system (Fig. 4b). Ag-NOR staining was noted in the interstitial region of the long arm of pair 12 (Fig. 4b, box). CH was found in the pericentromeric regions of most chromosomes, and large CH blocks were found in the proximal regions of the long arm of pairs 3, 4 and 9. Pair 1 had a heteromorphism in both males and females, probably because of a heterochromatin block, as did pair 12 (Fig. 4c). DAPI banding was positive in the CH regions, suggesting that these regions are AT-rich (Fig. 5a). CMA_3_ banding showed size differences between the homologs, suggesting the presence of a size difference in this GC-rich region (Fig. 5b). Finally, FISH against the 18S rDNA hybridized to the same region that was positive for Ag-NOR staining (Fig. 5c).

## Discussion

Both *Rhamphichthys marmoratus* and *Rhamphichthys* prope *rostratus* had 2n = 50, but differed in their KFs, with *Rhamphichthys marmoratus* having 44m/sm+6a and *Rhamphichthys* prope *rostratus* having 42m/sm+8a. Previously, *Rhamphichthys hanni* was described as having 2n = 50, but 20m+24sm+6a (Mendes et al. 2012). These differences can be explained by chromosome rearrangements that have altered the chromosome morphology but not the diploid number (e.g., pericentric inversions). These rearrangements can be sufficient to act as a post-mating reproductive barrier (King 1993). A more refined analysis, such as the use of chromosome painting, will be necessary for the precise determination of the rearrangements that differentiate the karyotypes of these three species. In a similar situation in Gymnotiformes, Nagamachi et al. (2010) demonstrated that two cytotypes of *Gymnotus carapo* Linnaeus, 1758 (2n = 42 and 2n = 40) differed not just by the fusion event suggested by the conventional analysis, but also by many rearrangements.

The CH in *Rhamphichthys* prope *rostratus* and *Rhamphichthys marmoratus* is AT-rich (i.e., DAPI banding-positive), which is consistent with other species of Gymnotiformes (Milhomem et al. 2007, 2008, Silva et al. 2008, Silva et al. 2009). The CH blocks found in pairs 4 and 12 of *Rhamphichthys marmoratus* and in pairs 3, 4 and 9 of *Rhamphichthys* prope *rostratus* can be used as cytogenetic markers for these species, as suggested for other Neotropical fish species (Almeida-Toledo 1998, Silva et al. 2008). Mendeset al. (2012) found only three submetacentric pairs with heterochromatin blocks in *Rhamphichthys hanni*. This is an important trait and can be used along with other characteristics to differentiate populations of these species, since there is some debate regarding their interspecific boundaries.

The NOR was found on a secondary constriction and stained positive with CMA_3_ as previously observed on other species (Pendáset al. 1993, Fernandes et al. 2005, Milhomem et al. 2007, Silva et al. 2008, De Souza et al. 2009). Each of the species studied herein had a single NOR, but *Rhamphichthys* prope *rostratus* had a size heteromorphism in this region. The 18S rDNA probe hybridized to a similar-sized segment in both homologs, suggesting that the size difference is not likely to be the result of an in-tandem duplication of the ribosomal genes (Martins-Santos and Tavares 1996), as described in *Eigenmannia* sp.1 by Almeida-Toledo et al. (1996). Instead, the heteromorphism found by CMA_3_ banding can be explained by a variation in the amount of GC-rich sequences interspersed among the ribosomal genes in this region. In *Rhamphichthys hanni* (Mendes et al. 2012), the results of the Ag-NOR staining and 18S rDNA probe hybridization were very similar to our findings in *Rhamphichthys rostratus*.

The phylogeny proposed by Albert (2001) places the families Rhamphichthyidae and Hypopomidae into a monophyletic group (Rhamphichthyoidea) that is only distantly related to the clade that joins the families Sternopygidae and Apteronotidae. The monophyly of Rhamphichthyoidea was supported by the synapomorphic characteristics described by Triques (2005).

However Alves-Gomes et al. (1995) suggested that Hypopomidae is not monophyletic, in that the genera *Hypopygus* and *Steatogenys* are more closely related to Rhamphichthyidae. The cytogenetic data described herein, as well as the recent work of Cardoso et al. (2011), seem to support the latter phylogenetic arrangement, since all the *Rhamphichthys* karyotypes described to date have 2n = 50. Among the Hypopomidae, *Hypopygus* and *Steatogenys* have 2n = 50, but all of the other genera have lower diploid numbers (2n = 26 to 42, Table 2). However, while the *Rhamphichthys* have karyotypes with KFs similar to those of *Hypopygus* and *Steatogenys* (42-44 bi-armed and 6- 8 mono-armed chromosomes) the KFs diverge considerably into *Steatogenys*, ranging from all bi-armed chromosomes (*Steatogenys duidae*) to mostly mono-armed chromosomes (*Steatogenys elegans*). Conversely, the karyotype of *Hypopygus* has a KF similar to those of *Rhamphichthys*. These differences seem to indicate that the genera *Hypopygus* and *Steatogenys* split from *Rhamphichthys* at an earlier date than the *Rhamphichthys* species split from one another, which is consistent with the phylogeny of Alves-Gomes et al. (1995). The chromosome similarity between *Hypopygus* and *Rhamphichthys* suggests that these genera separated more recently than *Steatogenys*, or that chromosome evolution proceeded more quickly in the latter genus, with a buildup of autoapomorphies.

The available cytogenetic information on Gymnotiformes may be sparse (of eight species of this genus, only three have had their karyotypes analyzed), but the existing data show an important variability in this group. More cytogenetic investigations on the family Rhamphichthyidae are warranted, as they will help us better understand the chromosomal evolution of these fishes for use in other fields of science, and assist us in defining the boundaries of the *Rhamphichthys* species.
